# Missed Doses and Missed Appointments: Adherence to ART among Adult Patients in Uganda

**DOI:** 10.1155/2013/270914

**Published:** 2013-01-14

**Authors:** C. Shumba, L. Atuhaire, R. Imakit, R. Atukunda, P. Memiah

**Affiliations:** Uganda Program, Institute of Human Virology, University of Maryland, 124 Luthuli Avenue, Bugolobi, Kampala, Uganda

## Abstract

*Background*. Missed doses and appointments are predictors of incomplete adherence among patients on ART. The AIDSRelief model emphasizes treatment preparation and continuous treatment support for ART patients including community followup. *Methods*. In August 2008, a survey was conducted among patients on antiretroviral therapy (ART) (interquartile range for duration of ART = 29–46 months, median = 33 months, *n* = 763, age >16 years), in 15 health facilities in Uganda. Missed doses and appointments among adult patients on ART and the factors most associated with these were identified. Reasons for missed doses were also explored. *Results*. The survey revealed that 97% of the patients had not missed their doses in the last week while 93% had not missed their appointments in the last three months. For those who had missed their doses, the most common reasons were travel (48%) and forgetfulness (28%). There was a significant association between missing doses and missing appointments (*P* = 0.0004) and between alcohol use and missed doses (*P* < 0.005). *Conclusions*. The level of adherence to medication and clinic appointments for patients on ART in the study population was very high. It is important to strengthen adherence strategies at both facility and community levels to assist patients that are likely to miss their doses or appointments.

## 1. Background

Uganda has more than 1.2 million people living with HIV infection and in 2010 more than 120,000 got infected with HIV [1]. Although there are various efforts to scale up AIDS treatment, many HIV-infected people are unable to access treatment and the increasing new HIV infections still exceed the government's capacity to provide treatment [2]. While attempts are being made to reduce new infections and to scale up antiretroviral therapy (ART), it is equally important to improve adherence to regimens among the few who are already accessing ART for successful treatment [3]. ART delays progression to AIDS and death, hence, improved the quality of life for people living with HIV [4, 5]. Maintaining good adherence has been found to be difficult to achieve and sustained by both patients and health care providers [6, 7]. A major concern with the use of ART is the emergence of viral resistance, which is mainly due to insufficient compliance [8].

Different tools have been used to determine ART adherence rates and on average the rate of adherence to ART is about 70% [9]. A meta-analysis found adherence rates for African patients to be approximately 77% [10]. A study by Oyugi et al., that assessed correspondence between multiple measures of adherence and viral load suppression, found that the mean adherence was 91–94% by all measures [11]. Patients' adherence is dynamic either improving or declining over time. A prospective study conducted in southwest Ethiopia showed that adherence had declined by 3% by the third month of follow-up visit compared to the results of the baseline study at month zero [6]. ART is a long-term treatment, and sustaining acceptable level of drug adherence poses a challenge for both the patient and health care workers [12]. Therefore, the benefits of ART can only be achieved if health providers realize that adherence is a dynamic process, complex, and cannot be predicted by only patient characteristics, but continuous monitoring and integration into regular clinic followup [6, 12]. Different studies have revealed predictors of adherence success. These are a combination of patient demographic and behavioral factors and disease characteristics, medication factors and health systems factors [6, 13–15]. 

The aim of this study is to describe the factors associated with incomplete adherence among adult patients on ART in 15 health facilities in Uganda.

## 2. Study Setting and Methods

The study was conducted in 15 faith-based and stand-alone HIV clinics that provide high-quality HIV care and treatment to rural and underserved communities in the northern, southern, central, eastern, and western regions of Uganda. The study setting is described in detail in another paper [16].

### 2.1. Data Collection

Data was collected using the AIDSRelief Adherence Survey that has been piloted, validated, and used in six African countries. The survey was designed to identify specific indicators that influence the adherence to antiretroviral therapy and the increase in quality of life. The study assessed missed clinic appointments, missed doses, and whether patients had ever run out of medication as the measures of adherence. A sample of 763 patient adherence surveys were done to assess disease, treatment knowledge and services received between August 2008 and March 2009. In August 2008, we conducted a survey of patients having been on ART (interquartile range for duration of ART = 29–46 months, median=33 months, *n* = 763, >16 years), in 15 facilities in Uganda. 

This survey was done as a part of a routine quality improvement program. The adherence survey questionnaire was administered by trained health staff. Informed consent forms were provided to the patients after full explanation from the health staff about the survey. The patients appended their signature indicating consent to participate in the survey. Each patient had the right to withdraw at anytime in the course of the survey. 

### 2.2. Data Analysis

Data were entered, cleaned, and coded in to a computer and analyzed using Stata version 11. Bivariate analysis was carried out to determine the statistical significant association between explanatory variables and the outcome variable. Chi-square tests and their *P* values at the level of significance of 5%, as well as confidence intervals (CI), were used to define statistical associations between explanatory variables and outcomes. 

## 3. Results

Out of the 763 patients who participated in this study, 50.5% were in the age group of 16–39 years. About 70% of the total study participants were females. Most (66.6%) of the patients were engaged in an income generating activity before starting on ART. Over half (54%) of the patients owned a home, and 31.6% were renting at the time of the survey. Many of the patients (42.1%) had primary education and 38.4% had secondary education, while only 7.7% had attained university education. The 763 patients who participated in this study varied in age, gender, socioeconomic status, and education (Table 1).

The survey revealed that 97% of the patients had not missed their doses in the last week and 93% of patients had not missed appointments in the past three months. There was no significant association between the measures of adherence in this study (missed doses, missed appointments, and ever run out of medication) and the sociodemographic factors namely age, gender, and education level (Table 2). 

Out of the patients who reported ever missing their doses for the period on ART, the most common reasons were travelled, forgot, work conflict, and felt unwell (Table 3). 

This study found that patients on ART that missed their appointments (29.2%) were more likely to miss their doses compared to those that did not (5.7%). The findings are depicted in Table 4: *P* value = 0.0004, OR = 6.80, 95% CI (2.41–18.66).

Patients on ART who used alcohol were more likely to miss a dose during the course of their treatment compared to those who did not use alcohol (Table 5: *P* value <0.005 with CI 2.40–27.83). 

## 4. Discussion

This study found that patients on ART that missed their appointments were more likely to miss their doses compared to those that did not. While we explored the reasons for missed doses, we did not do the same for missed appointments, therefore, it is difficult to understand the reasons for missed appointments. However, what was clear is that missing a scheduled appointment is associated with missing ART doses. Other authors reported other common reasons responsible for nonadherence that are related to income and they include failure to get transport to reach ART clinics hence missing pharmacy refills, missed clinic appointments and missed doses [10, 17]. However, another study found that patients in developing countries can achieve good adherence despite limited resources [6]. A study by Byakika-Tusiime et al. [18] showed that in ART centers, where patients were required to pay user fees, their adherence levels were low while Orrell et al.found that low socioeconomic status was not a predictor of adherence for patients with fully subsidized therapy [19]. This clearly applies to this study context where all the patients despite their low income have good access to fully subsidized therapy and exhibited high adherence comparable to high income settings. Notwithstanding, it is important to consider the fact that some patients may lack the means to access the fully subsidized therapy due to poverty. In this way, poverty acts as an intervening variable in hindering access to the much needed lifesaving therapy. Regardless, there would be need to further understand the experiences of the patients who miss appointments in order to tailor interventions that best suit them. 

Our study was unique as it looked at patients who had been on treatment for a considerable amount of time. The study by Oyugi et al. followed up patients from baseline to 12 weeks [11] while in our study patient duration of ART was more than 2 years at least. Most studies have not considered patient adherence beyond 2 years after ART initiation [4, 12, 15].

Our study is also the first of its kind that has explored the risk factors associated with incomplete adherence among elderly HIV patients. We found that there was no difference in adherence between the elderly who made up 12.5% of the study population and other age groups. However, this could be due to the small number of patients with nonadherence events to allow for risk factor analysis across all age groups including the elderly. 

In our study, gender and disclosure were not associated with adherence. In contrast, another study found that women were more likely than men to forget to refill medications and not to know how to take the medications correctly [20]. A few studies have reported age as an influencing factor to adherence especially in infancy and adolescents [7, 18]. Generally however, it has been reported that sociodemographic factors seem not to predict adherence [6], although there are a few factors which have been reported to hinder adherence such as low income which has been cited as a strong predictor of failure to adhere to ART [4, 7, 10]. 

In general, patient self-reported adherence in this survey was very high (97%). Although self-reported outcomes of adherence may have a weakness of reliability, a study by Oyugi et al. found no significant difference between patient-reported and objective measures of adherence [11]. Our findings are higher than the average adherence rates for African patients [10] but consistent with those in a prospective observational cohort study conducted in Dakar, Senegal, where adherence was estimated to be over 91% and similar to the study by Oyugi et al. [11]. Our findings are consistent with follow-up cohorts that were done in developed countries such as the APROCO cohort study and the Ciel Bleu trial [4]. The high levels of adherence in this patient-population can be attributed to the model of HIV care and treatment that is used. The model emphasizes adherence as a therapeutic intervention with intensive treatment preparation, patient specific care and treatment plan, community-based treatment services with home-based followup during ART startup. Disclosure is highly encouraged and all clinics have well-defined catchment area as well as longitudinal medical records for all patients.

This study found an association between alcohol use and missed doses. This is similar to other studies where alcohol consumption has also been reported to affect adherence [21–23]. Alcohol use is known to be a proxy for loss of control, thus, affecting adherence. In a study done in Botswana, around 40% of the patients surveyed admitted missing a dose because of alcohol consumption [24]. Related studies also show that alcohol is greatly related to the reduced adherence [25, 26]. A systematic review conducted in 2009 revealed that HIV/AIDS patients that used alcohol were 50–60% not likely to adhere to their medications [27]. 

There was some association between the educational status and missed appointments in the last three months in this study (*P* < 0.1) although the population educational levels were generally very low. A systematic review conducted in 2006, revealed that education and literacy levels are important predictors of adherence [28]. This paper found out that patient understanding of their medication regimen and their knowledge of the relationship between nonadherence and buildup of resistance to medication also predict better adherence. A patient's belief and self-determination to therapy influence adherence to medication. It is not clear however, what the optimal educational level would be to get better adherence but perhaps what is more important is the patient understanding of adherence on the disease mechanism. 

While many studies have shown that disclosure is associated with adherence [7, 28, 29], we did not find this association. The rate of disclosure to a friend or a relative in our patient pool for this study was quite high (99%) since patients are encouraged to disclose their HIV status to a treatment buddy before starting ART in order to improve the social support [16]. It is very important for health workers to find out the patients' disclosure before administering ART, while continuing to support those who would not have disclosed [28]. 

Different studies have also revealed that when patients experience side effects of prescribed medication, they tend to stop treatment or take it irregularly [6, 18]. In this study, only one patient out of those who missed their doses reported missing medication due to side effects and another due to pill fatigue. Similarly, patients have reported taking drug holidays as a result of pill fatigue [28]. Additionally, pill burden has been found to contribute to poor adherence [4, 7, 28]. However, pill burden was not mentioned as a problem for the patients in our study.

A number of studies have reported on irregular drug supply as an important predictor of adherence to ART [28, 30]. As far as the researchers know, drug stockouts in this study's patient population have never occurred but it is definitely appreciated that erratic drug supply can interrupt patient adherence. Some researchers have even argued that health care-related challenges could be the most important barriers to ART adherence in resource-limited settings [31]. Patient-provider relationships, the time spent at health facility, and opening hours can influence adherence to ART [17, 31]. Future research will need to study how these factors may be viewed as affecting adherence in this setting. 

## 5. Conclusion

While the level of good patient adherence to ART in this study was generally high, it is important to continue to reinforce the importance of adherence. High adherence rates can be achieved if HIV clinics and providers reduce the number of missed appointments as this study has shown that this is an important predictor of adherence. The model of treatment and care used in this population group is worth emulating. The reasons for missed appointments need to be explored to target interventions appropriately. Most patients reported that travelling and forgetfulness were important predictors of missed doses in this study. Thus, creativity in collaboration with the patients is required to design strategies to reduce this. Additionally, treatment and care providers need to strengthen patient education and counseling on the negative impact of alcohol use on adherence. 

## Figures and Tables

**Table 1:** Demographic characteristics of the study population.

Demographic characteristics	Frequency (percentage)
Age	
16–39	384 (50.53)
40–50	281 (36.97)
>50	95 (12.50)
Gender	
Female	528 (69.5%)
Male	232 (30.5%)
Socioeconomic status	
Engaged in any income generating activity before starting ART	
No	258 (33.4)
Yes	514 (66.6)
Living situation	
Own home	417 (54.02)
Rent	244 (31.60)
Living with friends	1 (0.13)
Living with relatives	71 (9.20)
Others	39 (5.05)
Education status	
Primary	295 (42.1)
Secondary	269 (38.4)
University	54 (7.7)
Below primary school	83 (11.8)

**Table 2:** Factors linked to the three measures of adherence in this study.

Adherence measure	Independent variables	No	Yes	OR	*P* value
		Freq. (%)	Freq. (%)	(95% CI limits)	
(1) Ever run out of medication(*N* = 444)		418 (94.1)	26 (5.9)		

	Gender (*N* = 439)				
	Male	122 (92.4)	10 (7.6)	0.58 (0.24–1.46)	0.296
	Female	239 (95.4)	14 (4.6)		
	Age (*N* = 43)				
	16–39	205 (96.61)	14 (6.39)		0.656
	40–50	157 (95.15)	8 (4.85)		
	>50	53 (96.36)	2 (3.64)		
	Educational level (*N* = 419)				
	Below primary	56 (94.92)	3 (5.08)		0.629
	Primary	167 (95.98)	7 (4.02)		
	Secondary	145 (94.16)	9 (5.84)		
	University	29 (90.63)	3 (9.38)		

(2) Doses missed in the last week (*N* = 763)		739 (96.9)	24 (3.1)		

	Gender (*N* = 760)				
	Male	222 (95.7)	10 (4.3)	0.60 (0.25–1.49)	0.228
	Female	514 (97.3)	14 (2.7)		
	Age (*N* = 760)				
	16–39	370 (96.4)	14 (3.6)		0.694
	40–50	273 (97.2)	8 (2.8)		
	>50	93 (97.9)	2 (2.1)		
	Disclosed HIV status to sexual partner (*N* = 714)				
	No	426 (96.4)	16 (3.6)	0.60 (0.21–1.66)	0.402
	Yes	266 (97.8)	6 (2.2)		
	Educational level (*N* = 419)				
	Below primary	55 (93.22)	4 (6.78)		0.450
	Primary	170 (97.70)	4 (2.30)		
	Secondary	148 (96.10)	6 (3.90)		
	University	31 (96.88)	1 (3.13)		

(3) Appointments missed in the past 3 months (*N* = 763)		706 (92.5)	57 (7.5)		

	Gender (*N* = 760)				
	Male	217 (93.5)	15 (6.5)	1.00 (0.51–1.96)	0.989
	Female	494 (93.6)	34 (6.4)		
	Disclosed HIV status to sexual partner (*N* = 717)				
	No	415 (93.0)	31 (7.0)	0.95 (0.50–1.80)	0.995
	Yes	253 (93.4)	18 (6.6)		
	Age (*N* = 760)				
	16–39	363 (94.5)	21 (5.5)		0.540
	40–50	260 (92.5)	21 (7.5)		
	>50	88 (92.6)	7 (7.4)		
	Educational level (*N* = 419)				
	Below Primary school	56 (94.92)	3 (5.08)		0.079
	Primary	163 (93.68)	11 (6.32)		
	Secondary	143 (92.86)	11 (7.14)		
	University	26 (81.25)	6 (18.75)		

*The differences in *N* are due to nonresponse.

**Table 3:** Reasons for missed doses.

Reasons for missing doses	No	Yes
	Freq. (%)	Freq. (%)
Travelled (*N* = 58)	30 (50.8)	29 (49.2)
Forgot (*N* = 60)	43 (71.7)	17 (28.3)
Work conflict (*N* = 59)	57 (96.6)	2 (3.4)
Felt unwell (59)	57 (96.6)	2 (3.4)
Side effects (59)	58 (98.3)	1 (1.7)
Tired of taking medication (59)	58 (98.3)	1 (1.7)

**Table 4:** Relationship between missed appointments and missed doses.

Missed appointment	Missed doses		Total
	No	Yes
No	694 (94.3)	42 (5.7)	736
Yes	17 (70.8)	7 (29.2)	24

**Table 5:** Relationship between alcohol use and adherence.

	Adherence measure	Yes	No	OR	*P* value
		Freq.	Freq.	95% CI limits	
	Missing doses (*N* = 417)				
	Yes	9	6	OR = 7.92	**<0.01**
	No	64	338	CI (2.40–27.83)	
	Missing appointment (*N* = 417)				
Alcohol use	Yes	8	23	OR = 1.72	0.206
	No	65	321	CI (0.63–4.19)	
	Ever run out off medication (*N* = 417)				
	Yes	6	16	OR = 1.84	0.215
	No	67	328	CI (0.57–5.16)	
